# Artificial intelligence in rheumatology: days of a future past

**DOI:** 10.1093/rap/rkaf022

**Published:** 2025-04-18

**Authors:** Vincenzo Venerito

**Affiliations:** Rheumatology Unit, Department of Precision and Regenerative Medicine-Ionian Area, University of Bari ‘Aldo Moro’, Bari, Italy

When discussing artificial intelligence (AI) in medicine, there’s often a tendency to frame it as a technology of tomorrow. Yet, as we stand in 2025, we find ourselves living in what can only be described as ‘days of a future past’—a time where yesterday’s imagined future has become today’s reality, particularly in rheumatology. The pace of AI integration has been so swift that many developments we once projected for the 2030s are already available to consumers and eventually operational in today’s clinics. What we perceive as futuristic is, in fact, our present, making this an era where the line between anticipated future and current reality has become remarkably blurred.

The rapid integration of AI tools into medical practice has created a fascinating paradox: we are simultaneously living in the future we once envisioned while continuously redefining what the future might hold. The tools and capabilities that seemed like distant possibilities just a few years ago are now part of our daily clinical practice, transforming what was once considered science fiction into today’s standard of care (Table 1).

**Table 1.** Comparative analysis of artificial intelligence in historical expectations, current realities and future directions in rheumatology

Aspect	Historical expectations	Current realities	Future directions and challenges
**Telemedicine**	Limited tools for remote consultations, considered futuristic.	Sophisticated systems like MeFisto support disease activity monitoring remotely.	Advancing wearable AI technologies for seamless, real-time patient monitoring.
**Medical imaging**	Heavily dependent on manual interpretation by specialists.	AI-driven radiomics and CNNs detect subtle diagnostic variations effectively.	Widespread deployment of AI as a co-diagnostic tool integrated into everyday workflows.
**LLMs in clinical practice**	Conceptualized for knowledge aggregation and theoretical applications.	Practical use in extracting insights from EHRs and literature for evidence-based care.	Deploying secure, locally operated LLMs in hospitals to maintain patient data privacy, aligning with EU AI Act mandates on safety and transparency.
**Patient stratification**	Generalized categories based on conventional classifications.	AI clustering unveils specific subgroups and patterns in diverse populations.	Expanding precision medicine with predictive analytics and multi-omics approaches.
**Ethics and equity in AI**	Rarely addressed in discussions of AI's medical role.	Growing focus on mitigating biases and promoting equitable AI applications.	Developing robust ethical guidelines and governance frameworks, tackling challenges like the EU AI Act's implementation and ensuring fairness, transparency, and inclusivity in AI-powered healthcare.

AI: artificial intelligence; CNNs: convolutional neural networks; EHRs: electronic health records; LLMs: Large Language Models.

This special issue showcases groundbreaking developments that demonstrate how AI is actively transforming rheumatology. Starting with telemedicine, the innovative MeFisto system represents a remarkable breakthrough in remote patient assessment. This technology can evaluate disease activity in rheumatoid arthritis patients through sophisticated hand motion recognition, enabling accurate remote monitoring without requiring in-person visits [1].

The emergence of Large Language Models (LLMs) represents a paradigm shift in clinical informatics and decision support systems, offering unprecedented opportunities for knowledge synthesis in rheumatology. Their application to electronic health records enables systematic extraction of clinically relevant information from unstructured medical narratives, facilitating the identification of complex phenotypic patterns and disease trajectories. Furthermore, LLMs’ capacity to process and contextualize vast amounts of medical literature and clinical guidelines positions them as powerful tools for evidence-based practice, potentially reducing diagnostic delays and optimizing therapeutic decision-making in complex rheumatological conditions [2].

In the domain of medical imaging, the transformative impact of radiomics and convolutional neural networks (CNNs) could reshape diagnostic capabilities [3, 4]. Through sophisticated radiomics approaches and advanced CNN architectures, AI systems are showing promising potential to achieve diagnostic accuracy comparable to experienced rheumatologists in detecting radiographic changes. Early studies suggest these systems could excel at identifying subtle alterations in medical images that might be overlooked by human observers, potentially providing a valuable complement to clinical expertise. While many of these developments remain in research settings, they represent a promising direction for transforming how we might analyse and interpret medical imaging in rheumatology.

In disease monitoring and management, AI algorithms are revolutionizing our understanding of patient populations through unsupervised learning approaches [5]. These sophisticated clustering techniques are uncovering previously unknown patient subgroups, leading to more nuanced disease classification and personalized treatment strategies. By analysing vast amounts of clinical, molecular and imaging data without predetermined categories, these algorithms are revealing natural disease patterns and patient subtypes that challenge our traditional disease classifications. This data-driven approach to patient stratification is particularly valuable in complex rheumatic diseases, where heterogeneous presentations and varying treatment responses have long challenged clinicians.

However, this rapid evolution presents both opportunities and challenges, particularly in the realm of ethics. The implementation of AI in rheumatology raises critical ethical considerations that demand immediate attention. Issues of data privacy, algorithmic bias and equitable access to AI-powered healthcare tools must be carefully addressed. We must ensure that these technologies do not exacerbate existing healthcare disparities but instead help bridge them. The transparency and interpretability of AI algorithms in clinical decision-making are crucial—clinicians must understand how these systems reach their conclusions to maintain accountability and trust in patient care. While this progress is transformative, it also necessitates alignment with emerging regulatory frameworks like the European AI Act [6]. The act, which introduces a risk-based approach to AI, emphasizes safety, transparency and equity, ensuring that AI innovations in medicine adhere to ethical standards and safeguard patient data.

The rheumatology community stands at a critical juncture where AI has already transformed from a future possibility to a present reality. This transformation demands immediate adaptation in our medical education to prepare future rheumatologists for an AI-augmented landscape. Our responsibility extends beyond mere technology adoption—we must actively participate in developing and implementing these tools while critically evaluating their performance and establishing guidelines for appropriate use.

As we navigate this intersection of technology and medicine, our focus must be on leveraging AI to enhance, rather than replace, clinical judgment. The challenge lies in maximizing these technologies’ benefits while addressing crucial considerations of data privacy, algorithmic bias and equitable access. By actively engaging with these innovations while maintaining the essential human element of patient care, we can ensure these powerful tools serve our patients’ best interests.

The future we once imagined is no longer on the horizon—it is our present reality. Our success in this AI-augmented era will depend on how effectively we shape this transformation, ensuring that these technologies advance the quality and accessibility of rheumatological care for all patients.

## Data Availability

No new data were generated or analysed in support of this article.
